# Detection of Anti-*Leptospira* IgM Antibody in Serum Samples of Suspected Patients Visiting National Public Health Laboratory, Teku, Kathmandu

**DOI:** 10.1155/2016/7286918

**Published:** 2016-12-04

**Authors:** Krishna Prasad Dahal, Supriya Sharma, Jeevan Bahadur Sherchand, Bishnu Prasad Upadhyay, Dwij Raj Bhatta

**Affiliations:** ^1^Central Department of Microbiology, Tribhuvan University, Kirtipur, Nepal; ^2^Tribhuvan University Teaching Hospital, Kathmandu, Nepal; ^3^National Public Health Laboratory, Teku, Nepal

## Abstract

Leptospirosis is a globally distributed zoonosis with varied clinical outcomes and multiorgan involvement in humans. In this study conducted from July 2011 to December 2011, 178 serum samples from patients suspected of leptospirosis were tested by Panbio IgM ELISA at National Public Health Laboratory, Kathmandu, out of which 51 (28.65%) were positive for anti-*Leptospira* IgM antibody. Leptospirosis was more common in people in their 2nd and 3rd decades of their life which together comprised 56.86% of the total positive cases. Most of those tested positive were farmers followed by students and housewives. Both animal contact and water contact seemed to play significant roles in disease transmission. Symptoms were vague with the most common being fever, headache, myalgia, abdominal pain, vomiting, jaundice, and diarrhoea. Life style heavily dominated by agronomical and farming activities in Nepal is conducive to leptospirosis transmission. Leptospirosis seems to be a significant public health problem in Nepal but is underestimated. In resource poor countries like Nepal where laboratories performing MAT or maintaining cultures are rarely available, serological test like ELISA could well depict the scenario of the disease prevalence.

## 1. Introduction 

Leptospirosis is a worldwide zooanthroponosis caused by pathogenic species of* Leptospira* [1]. Approximately half of the pathogenic serovars belong to* L. interrogans* or* L. borgpetersenii* [2]. The disease is of protean manifestation with outcomes ranging from an undifferentiated febrile illness to life threatening conditions such as Weil's disease and severe pulmonary haemorrhagic syndrome. Mortality from severe forms of the disease is about 5–40% and prompt diagnosis and early administration of antibiotics are critical in saving lives [3, 4]. Pathogenic leptospires are wide spread in nature and are capable of surviving in both environment and renal tubules of the host harbouring them [5]. Many wild and domestic animals like rats, dogs, cattle, pigs, horses, and so forth are potential reservoirs of the causative spirochetes [6, 7]. Infection occurs through contact with wild or domestic animals or exposure to soil or water contaminated by their urine [8].

Leptospirosis is difficult to diagnose because of its broad spectrum of symptoms and multiorgan involvement [8, 9]. Culture needs prolonged time period of weeks while PCR requires special equipment, highly skilled personnel and has not been evaluated worldwide [10, 11]. Microscopic Agglutination Test (MAT) is restricted to laboratories that are capable of maintaining strains for preparations of live antigens [8, 12]. ELISA uses a broadly reactive genus specific antigen to detect anti-*Leptospira* IgM and is a popular surrogate to MAT [10, 13].

Leptospirosis is highly prevalent in Asia Pacific Region and outbreaks in developing countries are most frequently related to normal daily activities, overcrowding, poor sanitation, and climactic condition [2]. The disease continues to have a major impact on people living in urban and rural areas of developing countries with inestimable morbidity and mortality [14]. Epidemics have been reported in Sri Lanka in 2008 and in the Philippines in 2009 [9]. It has been reported to be rampant in southern, central, eastern, and western India where heavy monsoon, animal rearing, unplanned urbanization, and agrarian way of life predispose to the infection [15]. The geographical location, climactic condition, and rich fauna of Nepal seem to be suitable for survival of leptospires. Thus leptospirosis could be a significant public health problem in Nepal and this study is designed to depict the disease scenario in Nepal.

## 2. Materials and Methods

The study was designed as a descriptive cross-sectional study and was carried out from July 2011 to November 2011 at National Public Health Laboratory, Teku, Kathmandu.

### 2.1. Study Population

Patients referred to National Public Health Laboratory for a test on leptospirosis were included into the study. About 5 mL of blood from adults and 3 mL from children aged 5 years or younger were collected and serum separated for further serological testing. Any icteric sample or sample exhibiting haemolysis, lipaemia, or microbial growth was excluded from the study.

### 2.2. Data Collection

A standardized form was used, to collect information from patients suspected of leptospirosis. Information regarding demographic details (age, sex), clinical features (fever, onset day, chills, malaise, myalgia, arthralgia, retro orbital pain, headache, anorexia, nausea, vomiting, abdominal pain, bleeding jaundice, and meningitis), animal or water contact/exposure, and occupation was obtained. Any suspected patient who has been handling farm animals like cows and buffaloes or handling pet dogs or playing with them was considered to have an animal contact. Similarly any suspected patient who has been swimming/bathing, washing clothes, and fishing in the river or working in water logged field was considered to have water contact.

### 2.3. Serological Study

Serum samples were subjected to IgM capture ELISA (Panbio* Leptospira* IgM ELISA from Inverness Medical Innovations, Australia) to detect anti-*Leptospira* IgM antibody present in the samples. Those giving positive result were defined as laboratory confirmed leptospirosis.

### 2.4. Statistical Analysis

Data were analysed using SPSS version 17.0. Values were expressed as mean ± standard deviation. Chi-square analysis was carried out and value of significance used for all statistical tests was *p* < 0.05.

## 3. Results

Out of 225 serum samples from patients suspected of leptospirosis, 178 were fit for inclusion into the study out of which 51 (28.65%) were positive for anti-*Leptospira* IgM antibody. Among the positives, 33 (64.70%) were males and 18 (35.30%) were females but the difference was statistically insignificant (*p* value 0.986). Most of the patients were adults with a median age of 26.50. The youngest age showing positive result was 5 years and the oldest age was 60 years. Most of the positive cases clustered in the age groups 20–30 (35.29%) and 30–40 (21.57%) whereas the disease was less prevalent in either extremes of age (Table 1).

Patients having animal contact were 117 out of which 42 were positive for anti-*Leptospira* IgM and out of 80 patients traced to have water contact 30 were positive for anti-*Leptospira* IgM (Table 2).

58 patients have both animal and water contact while 39 patients have neither animal nor water contact. Both modes of contact were found to be significantly related to leptospirosis.

Symptoms were vague and varied with the most common being fever (100%), headache (78.43%), myalgia (68.63%), abdominal pain (31.37%), vomiting (23.53%), jaundice (17.65%), and diarrhoea (15.69%). Conjunctival suffusion, muscle tenderness, retro orbital pain, rashes, and meningitis could not be reported (Table 3).

Among the suspected cases most were farmers (32.02%) and students (32.02%) followed by housewives (16.29%) while among the positive cases 45.10% were farmers followed by students (29.41%) and housewives (13.72%). Six of the cases could not be ascribed to any occupation (Table 4).

## 4. Discussion

The study was conducted at National Public Health Laboratory, Teku, Kathmandu, on samples referred for a test on leptospirosis. Leptospirosis is not routinely diagnosed in Nepal and it is very likely that only the samples that could not be attributed to other febrile illnesses were sent for a test on leptospirosis. This fact combined along with small sample size is likely the cause of apparently high positivity of 28.65% compared to the finding that only 4.1% of febrile cases reported to Patan Hospital in 2001 were attributed to leptospirosis [16].

In our study males were more affected than females. This could be because of higher exposure of males to risk factors like animal rearing, working in fields, and others like swimming in the rivers. The preponderance of leptospirosis in males is in agreement with the finding of other workers [15, 17–19]. The clustering of the disease in people in their 20s and 30s looks reasonable as people from these age groups are more involved in agriculture and animal rearing and hence are more likely to be exposed to risk factors associated with leptospirosis.

In our study both animal and water contact were significantly associated with leptospirosis. This could be in part due to small sample size. Rearing cattle and/or buffaloes are a common practice in Nepal. These animals are often chronically colonized with pathogenic leptospires with frequent transmission to humans [2, 14, 20, 21]. The most common modes of water contact were working in the fields, swimming, bathing, and/or washing clothes in the rivers. Common source outbreaks have been reported following swimming in contaminated water [7]. Our study coincided with the rice growing season. The characteristics of water in the rice fields are appropriate for the survival of leptospires [7].

The symptoms complained were vague and varied with the most common being fever along with headache, myalgia, vomiting, jaundice, and diarrhoea. Other symptoms of interest like conjunctival suffusion, retro orbital pain, muscle tenderness, rashes, and meningitis could not be reported in our study which makes our analysis of clinical features insufficient to ascribe any symptom or combinations significantly to leptospirosis.

Most of those affected were farmers (45.10%) followed by students (29.41%) and housewives (13.72%). Leptospirosis has traditionally been considered a disease of farmers [14]. The study period coincided with rice growing season and occupational activities that require prolonged contact with water or mud are associated with greater risk of infection [17, 19]. Rearing cattle and/or buffaloes go hand in hand with agricultural practices as most of the manures needed for the crops are supplied by these animals, hence putting farmers and housewives in a greater risk. Students are likely to get exposed to risk factors while swimming and/or bathing in rivers or ponds and many also accompany their parents in fields for agricultural works. Housewives in Nepal are generally involved in animal rearing and agricultural works. Leptospirosis has been frequently found in students and housewives [15, 22].

The limitations of the study include the use of single serum sample for ELISA instead of paired sera and the short duration and small sample size. We could not perform MAT of ELISA positive samples which would have given knowledge of infecting serovars. However we believe that the result of this study depicts the general features of the disease in Nepal.

## 5. Conclusion

Life style heavily dominated by agronomical and animal rearing practices in Nepal is conducive to the transmission of leptospirosis. The disease seems to be a significant public health problem in Nepal but is underdiagnosed and underestimated. In resource poor countries like Nepal where laboratories performing MAT or maintaining cultures are rarely available, serological tests like ELISA can be helpful in early diagnosis of the disease.

## Figures and Tables

**Table 1:** Age wise distribution of leptospirosis.

Age group	Number of cases	Number of positives (%)	% in total ELISA positives	*p* value
0–10	14	1 (7.14)	1.96	
10–20	36	9 (25)	17.65	
20–30	54	18 (33.33)	35.29	
30–40	29	11 (37.93)	21.57	
40–50	21	7 (33.33)	13.73	0.83
50–60	11	3 (27.27)	5.88	
60–70	10	2 (20)	3.92	
70–80	2	0 (0)	0	
80–90	1	0 (0)	0	

Total	178	51 (28.65)	100	

**Table 2:** Animal and water contact pattern in leptospirosis.

Contact type	Number of cases	Number of positives (%)	% in total positives	*p* value
Animal contact	117	42 (35.90)	82.35	0.003
Water contact	80	30 (37.5)	58.82	0.018

**Table 3:** Clinical features in leptospirosis.

Clinical signs/symptoms	Number of cases among positives showing the symptoms	% of total positive Cases
Fever	51	100
Headache	40	78.43
Myalgia	35	68.63
Vomiting	12	23.53
Diarrhoea	8	15.69
Jaundice	9	17.65
Abdominal pain	16	31.37

**Table 4:** Occupation wise distribution of leptospirosis.

Occupation	Number of cases (% of total cases)	Number of positives (%)	% in total positive cases
Farmer	57 (32.02)	23 (40.35)	45.10
Student	57 (32.02)	15 (26.31)	29.41
Housewife	29 (16.29)	7 (24.14)	13.72
Business	15 (8.43)	2 (13.33)	3.92
Service	14 (7.86)	4 (28.57)	7.84
Unemployed	6 (3.37)	0 (0)	0

Total	178	51 (28.65)	100
